# EBV Negative Lymphoma and Autoimmune Lymphoproliferative Syndrome Like Phenotype Extend the Clinical Spectrum of Primary Immunodeficiency Caused by STK4 Deficiency

**DOI:** 10.3389/fimmu.2018.02400

**Published:** 2018-10-16

**Authors:** Cyrill Schipp, David Schlütermann, Andrea Hönscheid, Schafiq Nabhani, Jessica Höll, Prasad T. Oommen, Sebastian Ginzel, Bernhard Fleckenstein, Björn Stork, Arndt Borkhardt, Polina Stepensky, Ute Fischer

**Affiliations:** ^1^Department of Pediatric Oncology, Hematology and Clinical Immunology, Medical Faculty, University Children's Hospital, Heinrich-Heine-University, Düsseldorf, Germany; ^2^Medical Faculty, Institute of Molecular Medicine I, Heinrich-Heine-University, Düsseldorf, Germany; ^3^Department of Computer Science, Bonn-Rhein-Sieg University of Applied Sciences, Sankt Augustin, Germany; ^4^Department of Clinical and Molecular Virology, Friedrich-Alexander-University Erlangen-Nuremberg, Erlangen, Germany; ^5^Department of Bone Marrow Transplantation and Cancer Immunotherapy, Hadassah University Hospital, Jerusalem, Israel

**Keywords:** primary immunodeficiency, serine/threonine kinase 4 (STK4)-deficiency, autoimmune lymphoproliferative syndrome, lymphoma, epstein barr virus

## Abstract

Serine/threonine kinase 4 (STK4) deficiency is an autosomal recessive genetic condition that leads to primary immunodeficiency (PID) typically characterized by lymphopenia, recurrent infections and *Epstein Barr Virus (EBV)* induced lymphoproliferation and -lymphoma. State-of-the-art treatment regimens consist of prevention or treatment of infections, immunoglobulin substitution (IVIG) and restoration of the immune system by hematopoietic stem cell transplantation. Here, we report on two patients from two consanguineous families of Turkish (patient P1) and Moroccan (patient P2) decent, with PID due to homozygous *STK4* mutations. P1 harbored a previously reported frameshift (c.1103 delT, p.M368RfsX2) and P2 a novel splice donor site mutation (P2; c.525+2 T>G). Both patients presented in childhood with recurrent infections, CD4 lymphopenia and dysregulated immunoglobulin levels. Patient P1 developed a highly malignant B cell lymphoma at the age of 10 years and a second, independent Hodgkin lymphoma 5 years later. To our knowledge she is the first STK4 deficient case reported who developed lymphoma in the absence of detectable EBV or other common viruses. Lymphoma development may be due to the lacking tumor suppressive function of STK4 or the perturbed immune surveillance due to the lack of CD4+ T cells. Our data should raise physicians' awareness of [1] lymphoma proneness of STK4 deficient patients even in the absence of EBV infection and [2] possibly underlying STK4 deficiency in pediatric patients with a history of recurrent infections, CD4 lymphopenia and lymphoma and unknown genetic make-up. Patient P2 experienced recurrent otitis in childhood, but when she presented at the age of 14, she showed clinical and immunological characteristics similar to patients suffering from Autoimmune Lymphoproliferative Syndrome (ALPS): elevated DNT cell number, non-malignant lymphadenopathy and hepatosplenomegaly, hematolytic anemia, hypergammaglobulinemia. Also patient P1 presented with ALPS-like features (lymphadenopathy, elevated DNT cell number and increased Vitamin B12 levels) and both were initially clinically diagnosed as ALPS-like. Closer examination of P2, however, revealed active EBV infection and genetic testing identified a novel *STK4* mutation. None of the patients harbored typically ALPS-associated mutations of the Fas receptor mediated apoptotic pathway and Fas-mediated apoptosis was not affected. The presented case reports extend the clinical spectrum of STK4 deficiency.

## Introduction

Deficiency of serine/threonine kinase 4 (STK4), also referred to as mammalian sterile 20-like protein (MST1), is an autosomal recessive primary immunodeficiency (PID) typically characterized by profound CD4 lymphopenia and recurring infections (1–6). STK4 deficiency in humans leads to decreased proliferation, increased susceptibility to apoptosis and dysregulation of the transcription factor Forkhead box protein O1 (FOXO1) and its downstream targets in T cells (1–3). Leukocytes show defective adhesion and chemotaxis (5). Due to the T cell impairment, many STK4-deficient patients experience fulminant EBV infections leading to lymphoproliferation and lymphoma development (1, 2, 5). This EBV associated lymphoproliferation can be observed in various PIDs including T cell defects, e.g., in patients with mutations in the *Interleukin-2-Inducible T Cell Kinase (ITK)* gene or in the *Magnesium Transporter 1 (MAGT1)* gene (7–9). Other disorders associated with lymphoproliferation and EBV infections are X-chromosomal linked lymphoproliferative disorders (SAP deficiency, XIAP deficiency), CD27 deficiency or FAAP24 deficiency (10–14). Interestingly, a recent report showed that STK4 plays a critical role as a tumor suppressor in the development of lymphoma and leukemia (15), putting STK4 deficient patients at an even higher risk to experience malignancies. Here we report on two patients with primary immunodeficiency due to homozygous STK4 mutations that were both initially clinically diagnosed as potentially ALPS like. One of them presented with the first reported case of an EBV negative lymphoma in this patient cohort.

## Materials and methods

### Patients, relatives and healthy controls

Patients, relatives and healthy controls were enrolled in this study after obtaining written informed consent. All experiments were approved by the Ethical Review Boards of the Hadassah Hebrew University, the Israeli Ministry of Health and the University Hospital Düsseldorf.

### Isolation and cultivation of mononuclear cells

Peripheral blood was obtained from the patients, relatives and healthy individuals. Mononuclear cells (PBMC) were isolated using density gradient centrifugation and cultured in medium consisting of RPMI 1640 (Life Technologies, Darmstadt, Germany) and Panserin 401 (PAN-Biotech, Aidenbach, Germany) mixed 1:1, supplemented with 10% fetal calf serum (FCS, GE Healthcare, Little Chalfont Buckinghamshire, UK) and 100 μg gentamycin (Life Technologies) and 30 U/ml IL2 (Miltenyi, Bergisch Gladbach, Germany). For the first 4 days, cells were activated by addition of 7 μg/ml phytohemaggluttinine (PHA, Life Technologies).

### Transformation of primary T cells

T cells from patient P1 and healthy controls were immortalized by transformation with herpes virus saimiri as decribed (16).

### Whole-exome sequencing and bioinformatic analysis

To identify the disease causing mutations next generation sequencing was carried out after targeted enrichment of whole exonic regions from sheared genomic DNA of the patient and family members using the SeqCap EZ Exome Library 2.0 kit (Roche/Nimblegen, Madison, WI). 100 bp single-read sequencing was performed on a HiSeq2000 (Illumina, San Diego, CA). Sequencing data was aligned to the human genome assembly hg19 (GRCh37) using BWA (17). Sequencing data was converted using Samtools (18). Variation calls were obtained employing GATK, HapMap, OmniArray, and dbSNP134 datasets (The Broad Institute, Cambridge, MA). Single nucleotide variations were annotated using the Variant Effect Predictor, based on the Ensemble database (v70). Variations were imported into a proprietary MySQL database driven workbench (termed Single Nucleotide Polymorphism Database, SNuPy).

### Validation of the *STK4* sequence variations

Validation of *STK4* nucleotide variations were performed by PCR/Sanger sequencing using genomic DNA from the patients and family members. The following primers were used: *STK4* c.1103 delT (P1) forward: 5′-CCT TTT TCT AAT GCG CTG ATG-3′, reverse: 5′- ATC TTT TCC TGG GGT TCA GG-3′; *STK4* c.525+2 T>G (P2) forward: 5′- GGA CCT GAA TAA GTG TTA AAT CTC G−3′, reverse: 5′- TAA GCC TGC ATG AAC CAT GA−3′. DNA fragments were amplified by PCR employing the Phusion High Fidelity PCR Master Mix (NEB, Ipswich, MA), 0.5 μM each primer and 20 ng of template genomic DNA. Cycling conditions: 30 s at 98°C followed by 30 cycles of 7 s at 98°C, 23 s at 60°C, 30 s at 72°C and a final extension of 10 min at 72°C. Sanger sequencing was carried out by a core facility (BMFZ, University Düsseldorf, Germany). The nucleotide variations were visualized using sequencher software (Gene Codes, Ann Arbor, MI).

### Real-time PCR

cDNA preparation was carried out using the QuantiTect Reverse Transcription Kit (Qiagen, Hilden, Germany) according to the manufacturer‘s instructions. Quantitative Real Time PCR was performed using the QuantiFast SYBR Green PCR Kit (Qiagen) on a CFX Connect machine (Bio-Rad Laboratories GmbH, Munich, Germany) under the following conditions: an initial step of 95°C for 15 min was followed by 40 cycles of 94°C for 15 s, 55°C for 30 s, and 72°C for 30 s. For STK4, Bcl-2, IL-7RA, Foxo1, and GAPDH we used the QuantiTect Primer Assay (Qiagen, QT00068922, QT00025011, QT00053634, QT00044247, QT00079247). Expression was calculated as fold of control using the ΔΔCt method.

### Immunoblotting

Cells were lysed in RIPA buffer containing 1% NP40, 50 mM Tris, pH 7.5, 350 mM NaCl, 0.5 mM EDTA, 2 mM dithiothreitol, protease- and phosphatase- inhibitor cocktail. Proteins were separated on 8.5% polyacrylamide gels, transferred to nitrocellulose membranes and detected by chemiluminescence. The following antibodies were used: STK4 (#3682, Cell Signaling Technology, Leiden, Netherlands), β-Actin (#A2228, Sigma-Aldrich, Taufkirchen, Germany), anti-rabbit and anti-mouse antibody (both from Cell Signaling Technology, #7074S and #7076S).

### Cell proliferation

PBMC were treated with 7 μg/ml phytohemaggluttinine (PHA), 5 μg/ml anti-CD3 antibody (BioLegend, San Diego, USA) or 5 μg/ml tetanus toxoid (Sigma-Aldrich) and cultivated for 72 h. Radioactive uptake was quantified after 4 h priming with ^3^H-thymidine (Perkin Elmer, Rodgau, Germany). Primary T cells from patient P1, family members and healthy control were stimulated with the human T Cell Activation/Expansion Kit (Miltenyi) according to the manufacturer‘s protocol for up to 4 days. Every day cell count was analyzed with trypan blue (Sigma-Aldrich) solution to control cell proliferation over time.

### Apotosis

Activated primary or transformed T cells were stimulated with recombinant Fas ligand (Super-Fas, 50 ng/ml, Enzo Life Sciences, Loerrach, Germany) or 0.5 μM staurosporine (LC Laboratories, Woburn, MA) for 16 h or serum starved for 2 days or left untreated. Apoptosis was analyzed by flow cytometric measurement of Annexin V-FITC (BD Biosciences, Heidelberg, Germany) and propidium iodide (PI, Sigma-Aldrich) staining using a FACSCalibur equipped with CellQuest software according to the manufacturer's instructions (BD Biosciences).

## Background

We report on two patients. Patient P1, a girl, was born to a consanguineous family of Turkish origin. She had a history of pulmonary valve stenosis, recurring otitis and polyarthritis with massively elevated rheumatoid factors. At the age of 10, she presented in our department to exclude a rheumatoid disorder. Hematological studies showed CD4 lymphopenia with a profound dysregulation of immunoglobulin levels (decreased IgG, increased IgM and IgA). In addition, sonography revealed abdominal masses in the liver and enlarged lymph nodes, which were caused by a highly malignant non-classical B cell lymphoma with intermediary characteristics of diffuse large B cell lymphoma and Burkitt's lymphoma. Diagnostic for infectious disease in blood, liquor and a tumor biopsy returned negative for viral DNA (including CMV, EBV, HHV6, HSV1, and HSV2) and antibody titer analysis of the serum showed no sign of an active viral infection. In addition, immune histochemical analysis of tumor material demonstrated the absence of EBV positive blasts. She was then treated according to NHL-BFM 04 protocol (risk group 2) with an additional BFM-CC block (risk group 3, dosis reduced to 80%). She responded well and achieved a complete tumor remission fast. At the age of 12, she presented again with lymphadenopathy and suspicion of relapse, but pathohistological characterization showed no malignancy at that time. Because immunoglobulin dysregulation, CD4 lymphopenia and recurring infections persisted, targeted sequencing of the PID associated *activation-induced cytidine deaminase (AID)* and *uracil DNA glycosylase (UNG)* genes was carried out, but revealed no mutations.

Patient P2, a girl, was born to a consanguineous family of Arab origin. At the age of 5, she developed sudden onset of nasal bleedings. Complete blood count showed low platelet numbers and she was treated for immune thrombocytopenia with multiple courses of IVIG and steroids. At the age of 7, she developed recurrent chest infections with symptoms of malabsorption clinically diagnosed as cystic fibrosis. Sweat chloride tests showed a chloride level of 46 mmol/L. However, no mutations in the *cystic fibrosis transmembrane conductance regulator* (*CFTR)* gene were found, while a chest computed tomography scan revealed bronchiectasis and pulmonary nodules. The patient also experienced two hemolytic episodes (Hgb: 2.9 g/dL) with a positive direct coombs test. At the age of 14, she presented again at our ward with hematolytic anemia (Hgb: 4.29 g/dL). General examination showed clubbing of the fingers, lymphadenopathy and hepatosplenomegaly. Immunological characterization revealed CD4 lymphopenia, increased level of DNTs (13.2%) and hypergammaglobulinemia leading to the initial clinical diagnosis of ALPS like disease (19). But in addition and not characteristic for ALPS like patients she presented with fulminant EBV infection. After discharge, the patient did not return to our ward and died at the age of 15.

**Figure 1:**
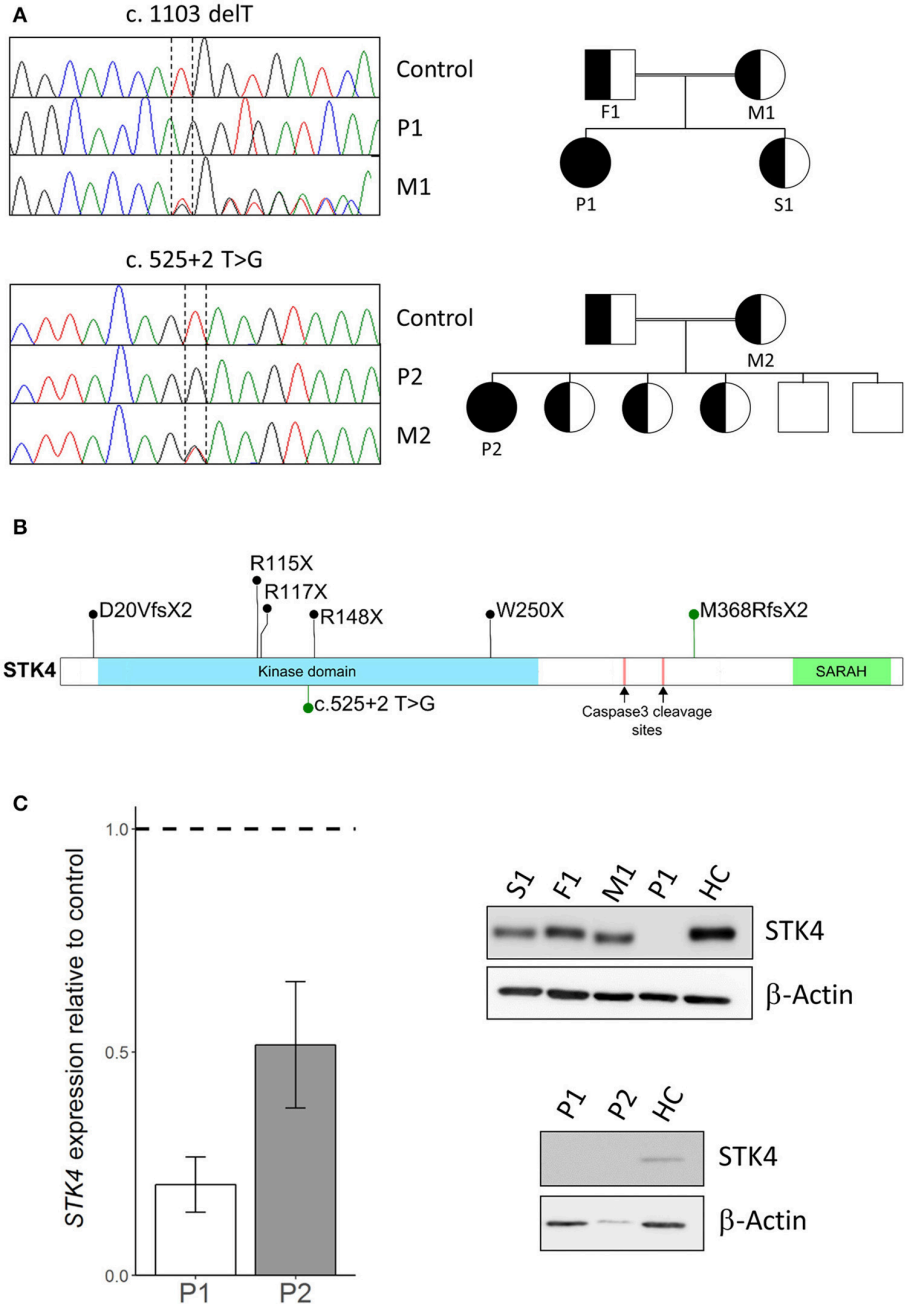
STK4 mutations in two patients. **(A)** Left*:* Sanger sequencing using genomic DNA confirmed homozygous *STK4* mutations in Patient 1 (P1) and Patient 2 (P2). Representative chromatograms of a healthy control, the homozygous patients (P1/P2) and the heterozygous mothers (M1/M2) of each patient are shown. Right*:* Pedigrees of the two consanguineous families are presented. The patients are the only diseased members of the family and the only homozygous mutation carriers. **(B)** Schematic drawing of the STK4 protein. The protein harbors a kinase domain and a SARAH (Sav/Rassf/Hpo) domain that mediates signal transduction and homodimerization of STK4. Two caspase cleavage sites are described that lead to truncation of the protein before the SARAH domain similar to the M368RfsX2 mutation of P1. Mutations identified in our patients are presented in green, other previously reported mutations in black [M368RfsX2, previously also described by (2)] (2, 3, 4, 5, 6). **(C)** Left*: STK4* transcript expression is reduced in both patients compared to healthy controls. Presented are levels of *STK4* mRNA expression in whole blood extracts relative to healthy controls assessed by qPCR. Expression was calculated as fold change compared to healthy control using the ΔΔCt method. GAPDH and β-actin expression were used as internal standards. A representative experiment of two is shown. Mean values of an experiment carried out in triplicates and corresponding standard deviations are shown. Right*:* STK4 protein deficiency is analyzed in patient derived transformed T cells of patient 1 and primary cells of patient 2. STK4 protein levels were analyzed by western blot. β-actin was used as a control. Total protein levels of P2 are low due to scarcity of primary patient material.

To identify the underlying genetic cause of the disease whole exome sequencing was performed on peripheral blood mononuclear cells (PBMC) of the patients and their family members. Next generation sequencing and data analysis revealed that both patients harbored homozygous *STK4* mutations segregated from their heterozygous parents. Patient P1 carried a previously described single nucleotide deletion leading to early protein truncation [c.1103 delT, p.M368RfsX2, (2)]. Patient P2 harbored a novel mutation at a splice donor site (c.525+2 T>G). Pedigree analysis revealed that the patients were the only homozygous mutation carriers in their families (Figure 1A). *STK4* mutations described in patients are homozygous nonsense or frameshift mutations that occur across the whole coding sequence. The M368RfsX2 that leads to truncation before the SARAH domain is the most C terminal described so far. The SARAH domain of STK4 is involved in signal transduction and homodimerization of STK4. The mutation found in P2 is the first splice site mutation reported in this cohort (Figure 1B). Expression analysis of STK4 on mRNA and protein level showed a decrease of *STK4* transcript levels in both patients compared to healthy controls and an STK4 protein deficiency for patient P1 (Figure 1C, Supplemental Figures 1, 2). Due to scarcity of patient material and lack of transformed cells from patient P2, the effect of the mutation on protein level is unclear. However, the mutation most likely leads to mis-splicing of STK4 within its kinase domain and any generated protein should be functionally deficient.

**Figure 2:**
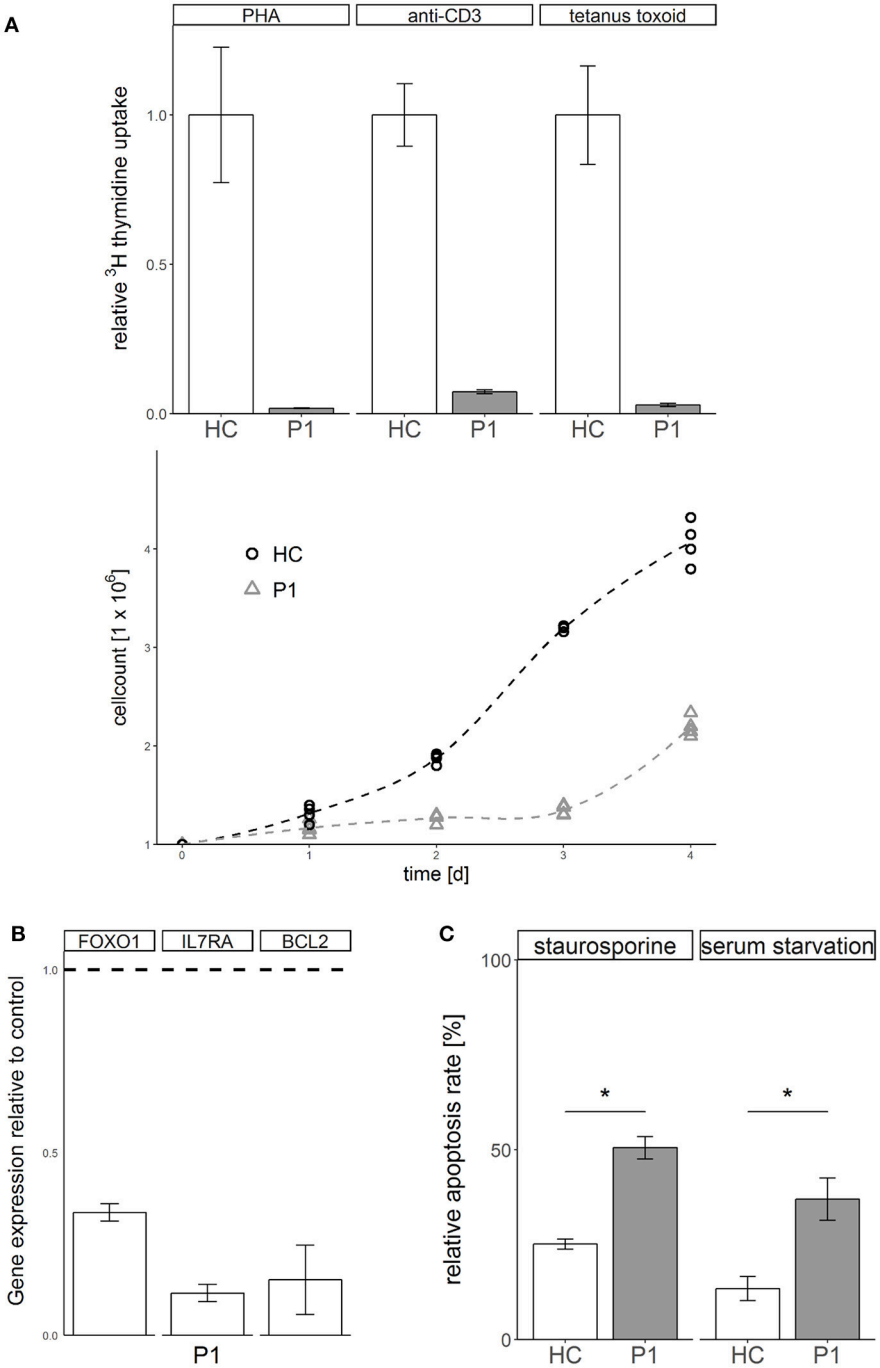
STK4 deficiency results in defective proliferation, gene dysregulation and increased susceptibility to apoptosis. **(A)** Proliferation is defective in STK4 deficient primary cells. Upper*:* Peripheral blood mononuclear cells of patient P1 and a healthy control were treated with the indicated lymphocyte activating compounds. Relative proliferation was quantified by ^3^H thymidine uptake (measured in counts per minute per 2 × 10^5^ cells) after 72 h. A representative experiment of two is shown. Relative values are given compared to a healthy control (HC, = 1). Mean values of triplicates and corresponding standard deviations are shown. Lower*:* Primary T cells of patient P1 and a healthy control were activated with PHA and cultivated in the presence of IL2. Viable cells were determined by trypan blue staining and counted with a Neubauer chamber. **(B)** STK4 deficiency dysregulates gene expression. Expression of target genes was determined in whole blood from patient P1 relative to healthy controls (dotted line). Expression was calculated as fold change compared to a healthy control using the ΔΔCt method. GAPDH and β-actin expression were used as internal standards. A representative experiment of two is shown. Mean values of an experiment carried out in triplicates and corresponding standard deviations are shown. **(C)** STK4 deficient T cells have a significantly increased apoptosis susceptibility in response to staurosporine or serum starvation compared to healthy control T cells. Primary T cells of patient P1 and a healthy control were stimulated with 0.5 μM staurosporine for 16 h or serum starved for 2 days or left untreated. Apoptosis was analyzed by flow cytometric measurement of Annexin V-FITC and PI staining. Relative apoptosis is presented compared to untreated controls. A representative experiment of two is shown. Mean values of an experiment carried out in duplicates and corresponding standard deviations are shown. *p < 0.05.

Functional analysis of primary patient derived cells from P1 showed a phenotype of impaired T cell proliferation (Figure 2A) and dysregulated gene expression of FOXO1, IL/RA, and BCL2 (Figure 2B). Transformed T cells derived from patient P1 showed increased susceptibility to apoptosis induced by kinase inhibitor staurosporine or serum withdrawal compared to healthy controls (Figure 2C). Not typical for ALPS like disease, these cells showed no defect of apoptosis induced via the Fas death receptor pathway (Supplemental Figure 3).

To investigate whether lacking or reduced *STK4* expression is a general trait of lymphomas, we analyzed the expression of STK4 in publicly available datasets of 201 healthy tissues and 1,675 lymphomas available in the R2: Genomics Analysis and Visualization Platform (http://r2.amc.nl). We found that STK4 was significantly downregulated in all lymphoma datasets compared to healthy datasets indicating a tumor suppressive role of STK4 (Supplemental Figure 4, Supplemental Table 1).

During further follow up patient P1 developed another EBV negative neoplasm, a Hodgkin lymphoma, at the age of 15 years. She received anti-CD20 monoclonal antibody rituximab and chemotherapy, but after one block of OEPA induction therapy (prednisone, doxorubicin, vincristine and etoposide as recommended by the EuroNet-PhL-C2 protocol) she experienced an infection or reactivation of Varicella zoster virus (VZV or human herpesvirus 3, HHV-3) and developed clinical herpes zoster with paraparesis and osteonecrosis of the legs. After treatment with cyclophosphamide and IVIG the neurological situation improved. Consolidation treatment with two modified COPDAC blocks (typically a combination of prednisone, vincristine, cyclophosphamide, and dacarbazine) was modified here by omission of prednisone and vincristine because of osteonecrosis and the neurologic situation. After achieving complete remission she received an allogeneic stem cell transplant from her HLA-matched sister. At the time of manuscript preparation, a few weeks after transplantation, she is tumor free and in good condition.

## Discussion

Homozygous *STK4* mutations have been described in PID patients presenting with CD4 lymphopenia and recurring infections (1–6). Here, we report on two additional patients with STK4 deficiency, one of them (P2) harboring the first homozygous splice site mutation (c.525+2 T>G) described in this patient entity and the other carrying a previously reported homozygous frameshift mutation (P1; c.1103 delT, p.M368RfsX2) (Table 1) (2). The initial clinical picture of these two cases resembled the phenotype of ALPS like syndromes (lymphadenopathy, increased numbers of DNT cells). This was more exlicit in the case of patient P2 who presented with classical clinical ALPS symptoms including lymphadenopathy, hepatosplenomegaly, highly elevated DNT cell number (13.2%), hemolytic anemia, and hypergammaglobulinemia. However, further clinical work up showed that, not typical for ALPS patients, these symptoms were associated with malignant disease at early age in patient P1 and with EBV infection in patient P2.

**Table 1:** Clinical and immunological characteristics of the patients.

	**P1**	**P2**	**Reference values**
STK4 mutation	c.1103 delT, p.M368RfsX2	c.525+2 T>G	
Age at first presentation	10	5	
Sex	Female	Female	
Hematology	Lymphadenopathy, CD4 lymphopenia, elevated DNT cell number, dysregulated Ig levels, increased Vitamin B12	Lymphadenopathy, hepatosplenomegaly, elevated DNT cell number, CD4 lymphopenia, thrombocytopenia, hemolytic anemia, hypergammaglobulinemia	
Infection	Recurring otitis, VZV/HHV3 infection/reactivation during chemotherapy, negative for CMV, EBV, HHV6, HSV1, and HSV2	Recurrent chest infections, active EBV infection	
Other clinical findings	B cell lymphoma, Hodgkin lymphoma, pulmonary valve stenosis, polyarthritis	Nasal bleedings, bronchiectasis and pulmonary nodules, clubbing of the fingers	
Treatment	1st lymphoma: NHL-BFM 04 protocol (risk group 2), additional CC block (risk group 3, dosis reduced to 80%), 2nd lymphoma: rituximab and chemotherapy: one OEPA and two modified COPDAC blocks, allogeneic hematopoietic stem cell transplantation of matched sibling donor	IVIG, steroids	
**IMMUNOLOGICAL PHENOTYPE**			
Leukocytes/μL	7400	na	4.1–8.3 × 1000/ μL
Lymphocytes [%]	6	25	34.5–48.2
Lymphocytes absolute	444	na	1.0–5.3 × 1000/μL
CD3+ [%]	71	80	52–78
CD3+CD4+[%]	10	18	25–48
CD3+CD8+[%]	50	50	9.0–35
CD45RA+CD4+[%]	30	na	49.3–72
CD45RO+CD4+[%]	70	na	24.5–44.4
CD45RA+CD8+[%]	48	na	62.3–86.3
CD45RO+CD8+ [%]	52	na	12.2–27.2
TCRαβ+CD4-CD8-[%]	3.7	13.2	< 2
CD45+CD127+ (IL7RA) [%]	8.7	na	65.8–89.6
CD20+ [%]	12	na	9.1–21
IgD+CD27-[%]	86	na	9.1–21
IgD+CD27+[%]	6	na	0.85–2.53
IgD-CD27+ [%]	2.4	na	4.1–18.7
CD3-CD56 + [%]	19.7	3	6.0–27
IgG [mg/dL]	147	1814	572–1474
IgA [mg/dL]	504	486	34–305
IgM [mg/dL]	752	33	31–208
Vitamin B12 [pg/mL]	>2000	na	197–866

*Cell surface markers were measured and analyzed by flow cytometry on a FACSCalibur using CellQuest software (Becton Dickinson Biosciences, Heidelberg, Germany). Immunoglobulins (Ig) were measured in the serum by ELISA. [CMV, Cytomegalovirus; DNT, double negative T cells; EBV, Epstein Barr virus; HHV3, human herpes virus 3; HHV6, human herpes virus 6; IVIG, intravenous immunoglobulins; NHL-BFM, Non-Hodgkin Lymphoma-Berlin-Frankfurt-Münster, VZV, varicella zoster virus (synonymous for HHV3)]*.

The cohort of STK4 deficient patients is highly susceptible to EBV infections. Eight of 15 reported cases so far presented with persistent EBV viremia and five of 15 developed EBV associated lymphoproliferative disorders, primarily affecting B cells (Table 2). The impact of active EBV infections on lymphoma development is well-established (20). Therefore, similar to other related disorders associated with EBV infections (such as MAGT1 or ITK deficiency), the lymphoma incidence in STK4 deficient patients is assumed to be increased although the absolute number of reported cases, including the case presented here is still low (*n* = 3). Sherkat et al. reported on a patient who presented first with a B cell lymphoproliferative syndrome and developed later an EBV+ primary cardiac T cell lymphoma, a very rare pediatric malignancy (1, 6). Nehme et al. reported on a case who suffered from an EBV+ Hodgkin B cell lymphoma (2). The patient P1 presented here harbored the same mutation as described by Nehme et al. and developed two independent lymphomas in the course of follow up: an aggressive B cell lymphoma and a Hodgkin lymphoma. Strikingly, both lymphomas were negative for EBV tested in blood, liquor and tumor biopsies. A hit-and-run mechanism of virus infection seems therefore unlikely. This strongly suggests a cancer predisposition mechanism of STK4 mutations independent of EBV infections.

**Table 2:** Lymphoproliferation and malignancies reported so far in STK4 deficient patients.

**Patient- No**.	**Persistent EBV viraemia**	**Lymphoproliferation/malignancy**	**References**
1	Negative		(1)
2	Negative		(1)
3	Positive	B-lymphoproliferative syndrome/ EBV+ primary cardiac T-cell lymphoma	(1, 6)
4	Positive	EBV+ Hodgkin B-cell lymphoma	(2)
5	Positive	B-lymphoproliferative syndrome	(2)
6	Positive		(2)
7	Negative		(2)
8	Negative		(3)
9	Negative		(4)
10	Negative		(4)
11	Positive	Lymphoproliferative syndrome	(5)
12	Positive	Lymphoproliferative syndrome	(5)
13	Positive		(5)
14	Negative	EBV-B-cell lymphoma and Hodgkin lymphoma	Present study
15	Positive	Lymphoproliferative syndrome	Present study

The potential role of STK4 in tumorigenesis was investigated in various studies in mice. Conditional knockout of the mouse gene homolog *Mst1* in intestine or liver resulted in the generation of solid tumors at these sites (21, 22). Pathogenic upregulation of the Hippo signaling pathway was revealed as the tumor driver. Kim et al. (15) reported that *Mst1* knockout mice were prone to leukemia and lymphoma development after mutagen treatment or p53 deletion. Lymphomagenesis was not driven by hippo upregulation under these conditions, but by chromosomal instability. They analyzed publicly available microarray gene expression data of human acute lymphoblastic leukemia and various human lymphomas from the Oncomine database (www.oncomine.org) that showed a downregulation of *STK4* in these entities. In the present study, we analyzed publicly available datasets of 201 healthy tissues and 1,675 lymphomas available in the R2 platform. We could confirm a significant general downregulation of *STK4* in lymphomas derived from B- T- and NK cells. The mechanisms of STK4 in lymphoma suppression still need to be clarified and will most likely be complex due to the multiple essential functions of STK4 in e.g. apoptosis, autophagy, chromosome stability, cell cycle progression. A perturbed B cell function is indicated by reduced B cell numbers and hypogammaglobulinemia in some STK4 deficient patients. In addition, Dang et al. demonstrated impaired adhesive response to chemokines in patient-derived EBV transformed B cells (5). However, a comprehensive analysis of STK4 deficient B cell function is still lacking. Our own *in vitro* analyses confirmed the effect of STK4 deficiency on lymphocyte function and gene expression established in the previous works.

A perturbed immune surveillance due to CD4 lymphopenia has to be taken into account as an EBV independent mechanism that may promote lymphoma development. In this line, approximately 20% of patients with idiopathic CD4 lymphopenia also develop malignancies, lymphoma being the most common one. EBV infections were remarkably uncommon in this patient entity (~ 2%) (23). A similar cohort with CD4 lymphopenia is presented by patients with HIV infections. The risk of lymphoma in HIV patients is increased 60–200 fold for non-Hodgkin lymphoma and 8–10 fold for classical Hodgkin lymphoma compared to the healthy population (24).

Our data suggests that patients with STK4 deficiency should be under close lymphoma surveillance even in the absence of EBV infections. In addition, a possibly underlying STK4 deficiency should be suspected and tested in patients with a clinical history of recurrent infections, CD4 lymphopenia and lymphoma and unknown genetic make-up.

## Author contributions

CS: Performed laboratory work, designed research, analyzed data, and wrote the paper; DS and AH: Performed laboratory work, designed research, analyzed data, and participated in writing the paper; SN: Performed laboratory work, designed research, and analyzed data; PS, JH and PO: Provided clinical samples and analyzed clinical data; BF: Provided reagents, designed and supervised research; BS: Designed and supervised research, analyzed data; SG: Provided bioinformatic analyses; AB: Designed research and critically reviewed the paper; UF: Designed research, analyzed data, and wrote the paper.

### Conflict of interest statement

The authors declare that the research was conducted in the absence of any commercial or financial relationships that could be construed as a potential conflict of interest.
