# Supplementary material for: EBV Negative Lymphoma and Autoimmune Lymphoproliferative Syndrome Like Phenotype Extend the Clinical Spectrum of Primary Immunodeficiency Caused by STK4 Deficiency
Source: Front Immunol. 2018 Oct 16;9:2400. doi: 10.3389/fimmu.2018.02400 (PMC6198654; doi:10.3389/fimmu.2018.02400)
Supplement: Supplementary file 1 [file Data_Sheet_1.docx]

Supplementary Material

EBV Negative Lymphoma and Autoimmune Lymphoproliferative Syndrome Like Phenotype Extend the Clinical Spectrum of Primary Immunodeficiency Caused by STK4 Deficiency

Cyrill Schipp, David Schlütermann, Andrea Hönscheid, Schafiq Nabhani, Jessica Höll, Prasad T. Oommen, Sebastian Ginzel, Bernhard Fleckenstein, Björn Stork, Arndt Borkhardt, Polina Stepensky, Ute Fischer*

*** Correspondence:** Ute Fischer: ute.fischer@med.uni-duesseldorf.de

# Supplementary Figures and Tables

## Supplementary Figures


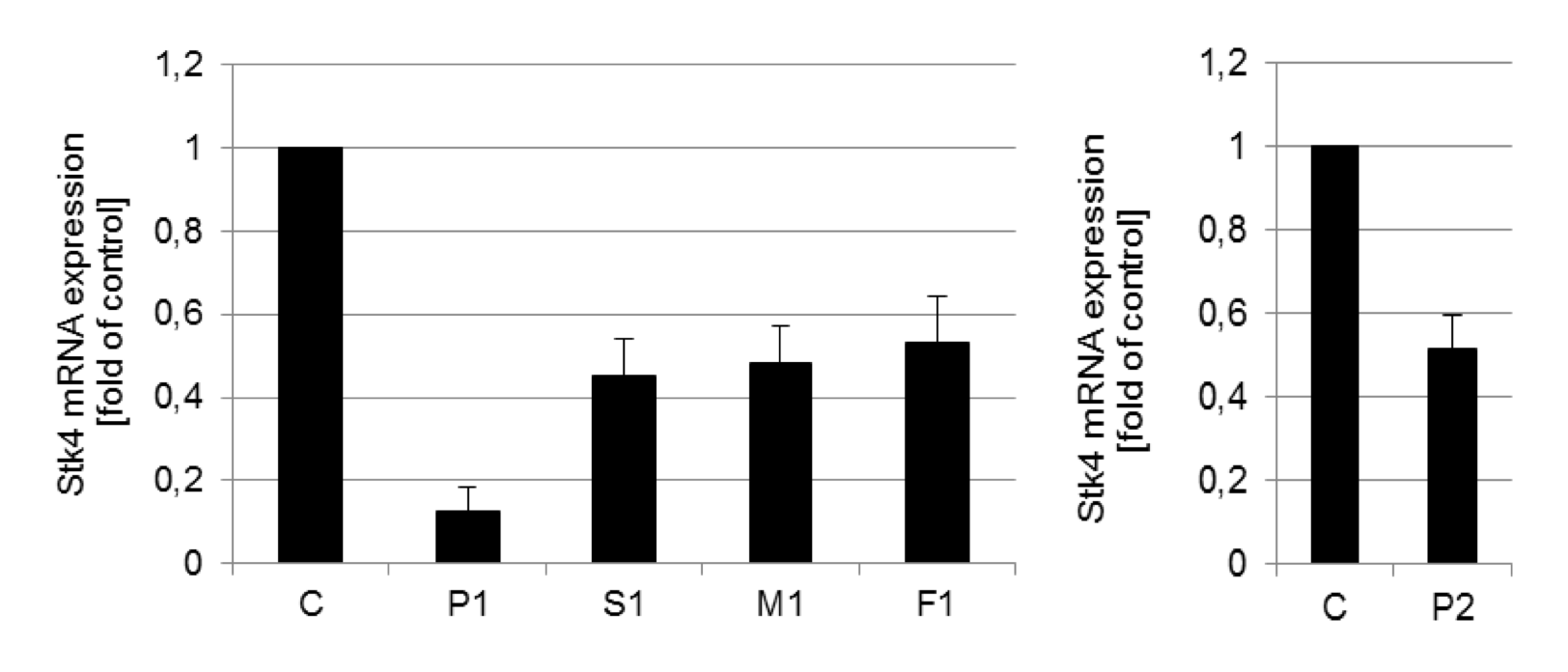


**Supplemental Figure 1.** STK4 transcript expression is reduced in both patients compared to healthy controls. Presented are levels of STK4 mRNA expression in whole blood extracts relative to healthy controls assessed by qPCR. Expression was calculated as fold change compared to healthy control (=1) using the ΔΔCt method. GAPDH and β-actin expression were used as internal standards. Mean values of representative experiments of two carried out in triplicates and corresponding standard deviations are shown.

**
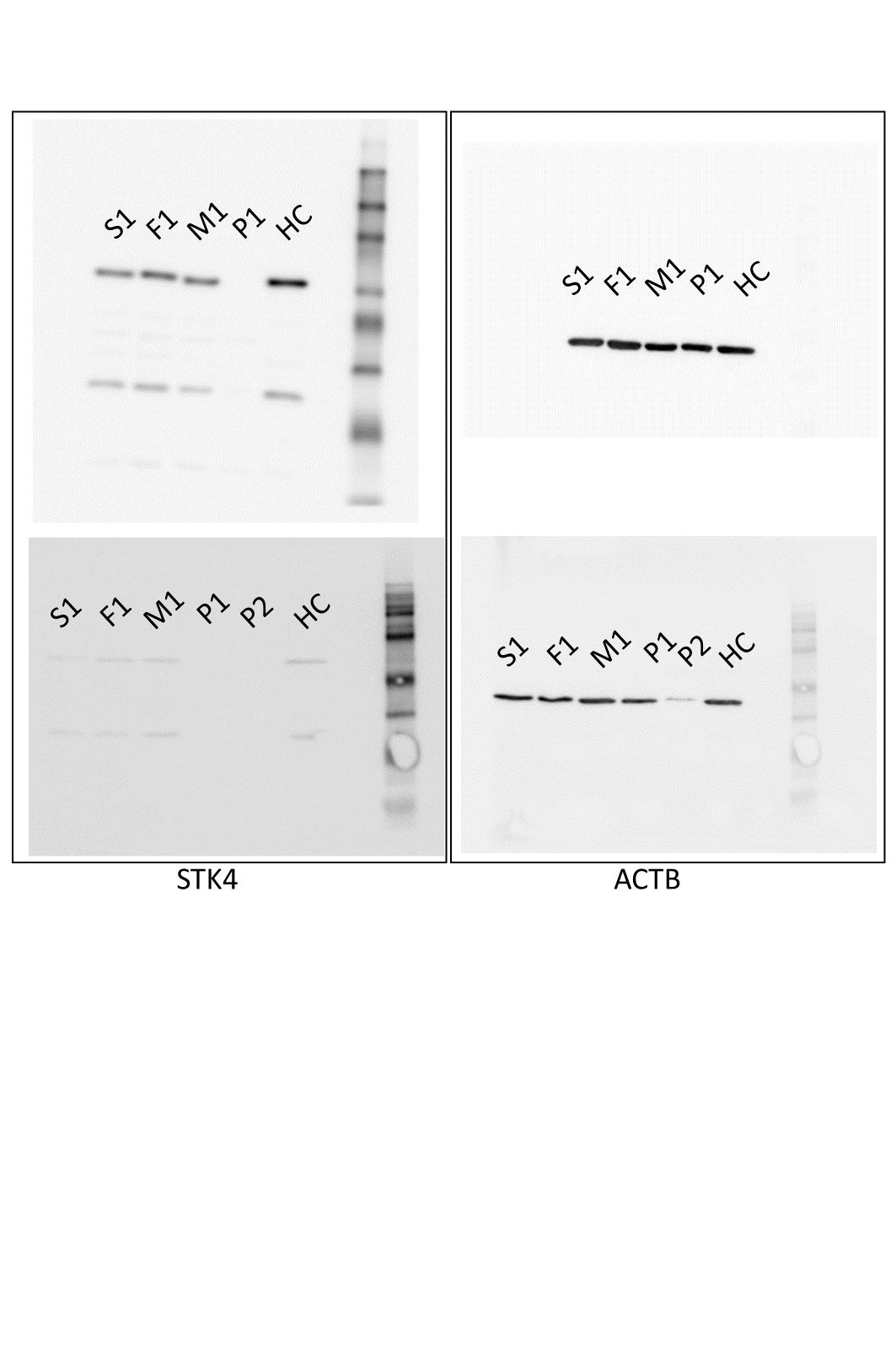
**

**Supplemental Figure 2.** The uncropped immunoblots used in Figure 1C are presented. HC: Healthy Control; P1: Patient 1; P2: Patient 2; M1: Mother of P1; F1: Father of P1; S1: Sibling of P1.

**
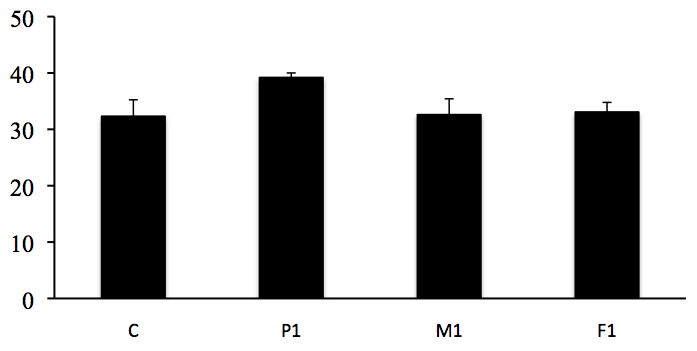
**

**Suplemental Figure 3:** Fas receptor mediated apoptosis is not significantly increased in patient P1. Activated primary T cells from a healthy control, the patient P1 and her father (F1) and mother (M1) were stimulated with recombinant Fas ligand (Super-Fas, 50 ng/ml, Enzo Life Sciences, Loerrach, Germany) for 16 hours or left untreated. Apoptosis was analyzed by flow cytometric measurement of Annexin V-FITC (BD Biosciences, Heidelberg, Germany) and propidium iodide (PI, Sigma-Aldrich) staining using a FACSCalibur equipped with CellQuest software according to the manufacturer’s instructions (BD Biosciences). Presented is the percentage of AnnexinV-FITC+/PI- cells. A representative experiment of three is shown carried out in duplicates. Mean values and standard deviations are shown.

**
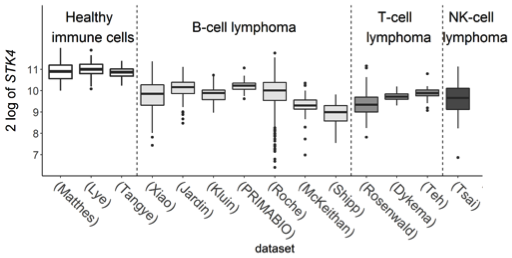
**

**Suplemental Figure 4:** STK4 expression in lymphomas**.** Analysis of *STK4* expression in datasets of 201 healthy tissues and 1675 lymphomas publicly available in the R2: Genomics Analysis and Visualization Platform (http://r2.amc.nl). Entities were grouped according to the major cell type in the lymphoma. Reduced expression in lymphoma datasets was highly significant compared to the tested healthy datasets (Supplement Table 1; T-test).

## Supplementary Table

|  | HC leukocytes  (Matthes) | HC lymphocytes  (Lyte) | HC PBMC  (Tangye) |
| --- | --- | --- | --- |
| HC lymphocytes (Lyte) | 1 | NA | NA |
| HC PBMC  (Tangye) | 1 | 1 | NA |
| B-cell lymphoma (Xiao) | 1,12E-19 | 3,09E-80 | 5,16E-08 |
| B-cell lymphoma (Jardin) | 3,93E-09 | 1,60E-36 | 1,73E-03 |
| B-cell lymphoma (Kluin) | 8,84E-11 | 1,21E-22 | 1,46E-05 |
| B-cell lymphoma (PRIMABIO) | 1,39E-06 | 1,08E-24 | 1,93E-02 |
| B-cell lymphoma (Roche) | 1,43E-17 | 3,67E-75 | 5,06E-07 |
| B-cell lymphoma (McKeithan) | 8,64E-25 | 1,37E-48 | 1,74E-13 |
| B-cell lymphoma (Shipp) | 1,00E-30 | 1,60E-50 | 1,04E-18 |
| T-cell lymphoma (Rosenwald) | 1,94E-34 | 4,17E-108 | 2,12E-15 |
| T-cell lymphoma (Dykema) | 7,70E-09 | 6,12E-15 | 2,63E-05 |
| T-cell lymphoma (Teh) | 5,64E-07 | 4,01E-12 | 4,35E-04 |
| NK-cell lymphoma (Tsai) | 4,76E-17 | 3,07E-35 | 5,73E-09 |

**Supplement Table 1: p-Values for supplemental Figure 4 are given.**
